# DED Interaction of FADD and Caspase-8 in the Induction of Apoptotic Cell Death

**DOI:** 10.4014/jmb.2206.06003

**Published:** 2022-07-25

**Authors:** Young-Hoon Park, Chang Woo Han, Mi Suk Jeong, Se Bok Jang

**Affiliations:** Department of Molecular Biology, College of Natural Sciences, Pusan National University, Busan 46241, Republic of Korea

**Keywords:** Apoptosis, FADD, caspase-8, DED, mutation, structure

## Abstract

Fas-associated death domain (FADD) is an adapter molecule that bridges the interaction between receptor-interacting protein 1 (RIP1) and aspartate-specific cysteine protease-8 (caspase-8). As the primary mediator of apoptotic cell death, caspase-8 has two N-terminal death-effector domains (DEDs) and it interacts with other proteins in the DED subfamily through several conserved residues. In the tumor necrosis receptor-1 (TNFR-1)-dependent signaling pathway, apoptosis is triggered by the caspase-8/FADD complex by stimulating receptor internalization. However, the molecular mechanism of complex formation by the DED proteins remains poorly understood. Here, we found that direct DED-DED interaction between FADD and caspase-8 and the structure-based mutations (Y8D/I128A, E12A/I128A, E12R/I128A, K39A/I128A, K39D/I128A, F122A/I128A, and L123A/I128A) of caspase-8 disrupted formation of the stable DED complex with FADD. Moreover, the monomeric crystal structure of the caspase-8 DEDs (F122A/I128A) was solved at 1.7 Å. This study will provide new insight into the interaction mechanism and structural characteristics between FADD and caspase-8 DED subfamily proteins.

## Introduction

In the receptor-dependent apoptotic signaling pathway (also called extrinsic apoptotic pathway), cell death is triggered by TNFR superfamily proteins that induce several signal transduction pathways controlling cell survival and death. DD superfamily proteins, including caspase-8, Fas-associated death domain (FADD), and receptor-interacting protein 1 (RIP1) play a crucial role in TNFR-1-dependent apoptosis (1-4). These proteins activate death signaling after TNFR-1 internalization. Adapter protein FADD directly binds to tumor necrosis factor receptor type 1-associated death domain (TRADD) or receptor-interacting protein 1 (RIP1) and activates an apoptosis-related cysteine peptidase caspase-8 [5, 6]. FADD interacts with several tumor necrosis factor receptor (TNFR) superfamily proteins to activate caspase-8 and subsequently induces cell death (7-10). Human FADD encodes a protein containing an N-terminal DED (death-effector domain) and a C-terminal DD (death domain) that regulate apoptosis and necrosis. FADD expression increases in cancer cells and tissues treated with anticancer agents, suggesting that FADD mediates the effects of these agents [11-14].

Caspases are a primary mediator of apoptotic cell death and are broadly categorized into initiator and effector caspase [15]. Initiator procaspase-8 death-fold motifs have two N-terminal DEDs that trigger apoptotic cell death by activating effector caspases [16]. The new paradigm of death complex proved that procaspase-8 is activated by the assembly of DED chains [17, 18]. These DED chains promote the homodimerization of procaspase-8 and are important for the caspase-dependent signaling cascade.

To elucidate the molecular mechanisms underlying the synergism between FADD and caspase-8, we investigated the interaction between recombinant FADD and caspase-8 DEDs. Direct DED-DED interaction between caspase-8 and FADD in vitro is demonstrated by several biochemical and biophysical assays. Moreover, we determined a monomeric crystal structure of caspase-8 (F122A/I128A) at 1.7 Å. The findings of this study could provide additional insight into the mechanisms underlying the interactions between DED superfamily proteins.

## Materials and Methods

### Expression, Purification, and Site-Directed Mutagenesis

The FADD DED (residues 1–83) and caspase-8 DEDs (residues 1–188) were subcloned into the His-tagged fusion protein vector pET-28a for purification. For soluble co-expression of the FADD/caspase-8 complex, FADD and caspase-8 DEDs were subcloned into pET-28a. In addition, the FADD DED (residues 1–83) was subcloned into a glutathione S-transferase (GST)-fused protein vector pGEX-4T-1 in order to perform the pull-down experiments. Double-stranded oligonucleotides were used to create nine mutations (I128A, R5E/I128A, Y8D/I128A, E12A/I128A, E12R/I128A, K39A/I128A, K39D/I128A, F122A/I128A, and L123A/I128A) in caspase-8 DEDs.

Histidine-tagged plasmids of the FADD and caspase-8 DEDs were transformed into overexpression competent cells [*E. coli* BL21(DE3)]. The caspase-8 DED-induced cell pellet was re-suspended in lysis buffer A [50 mM Tris-HCl (pH 7.5) and 200 mM NaCl] and FADD DED was re-suspended in lysis buffer B [50 mM Tris-HCl (pH 10.5), 200 mM NaCl, and 1 mM dithiothreitol (DTT), and 100 μM phenylmethanesulfonyl fluoride (PMSF)] with the protease inhibitor mixture. The soluble supernatants of His-tagged FADD and caspase-8 fusion proteins were purified using a Ni-NTA column and gel filtration chromatography. The GST-tagged FADD construct was transformed into the expression host *E. coli* BL21(DE3). The cell pellet was re-suspended with PBS buffer [4.3 mM Na_2_HPO_4_, 1.47 mM KH_2_PO_4_, 137 mM NaCl, and 2.7 mM KCl (pH 7.4)]. The supernatant of GST-tagged FADD fusion protein was loaded onto a Glutathione Sepharose 4 Fast Flow column (Sigma-Aldrich, USA) and the bound protein was eluted with buffer [50 mM Tris-HCl (pH 7.5)] containing 5-30 mM glutathione.

### Bio-Transmission Electron Microscope

A small drop of each protein in solution was placed on a formvar carbon-coated copper grid and negatively stained with 2% uranyl acetate for 1 min. The BIO-TEM image was recorded in a Tecnai G2 Spirit electron microscope (USA) equipped with a CCD camera at a magnification of 67,000X.

### GST Pull-Down Assay

For the GST pull-down assay, 50 μg of purified His-tagged proteins and their mutants were mixed with 50 μg of purified GST or GST-tagged proteins. Then, this was followed by incubation for 12 h at 4°C with gentle rotation. Pre-washed glutathione sepharose 4B bead was added with buffer A or PBS for 2 h at 4°C. The bead was centrifuged at 600 *×*g for 3 min and washed with buffer A or PBS. The protein bound to the bead was eluted [50 mM Tris-HCl (pH 7.5), 30 mM glutathione] and then resolved on a 15% SDS polyacrylamide gel. The protein was subsequently analyzed by western blot using anti-His or anti-GST.

### BIAcore Biosensor Analysis

Measurement of the apparent dissociation constant (*K*_D_) between FADD and caspase-8 DEDs was carried out using a Biacore T100 Biosensor (GE Healthcare Biosciences, Sweden). The purified FADD DED was covalently bound to the Series S sensor chip CM5 (carboxylated dextran matrix) using an amine-coupling method. The FADD DED (50 μg/ml) in 10 mM sodium acetate (pH 5.0) was coupled via injection for 15 min at 10 μl/min, followed by the injection of 1 M ethanolamine to deactivate residual amine. For kinetic measurement at 25°C, caspase-8 DED samples with concentrations ranging from 250 to 5,000 nM were prepared by dilution in HBS-EP+ buffer (10 mM of HEPES (pH 7.4), 150 mM of NaCl, 3 mM of EDTA and 0.05% v/v surfactant P20). The immobilized ligand was regenerated by injecting 50 mM NaOH at a rate 10 μl/min during the cycles.

### Crystallization

A crystal of the caspase-8 DEDs (residues 1–188, F122A and I128A) was obtained by the hanging-drop vapor diffusion method. Each drop of the caspase-8 DEDs in buffer A was mixed with a reservoir solution consisting of 20% w/v polyethylene glycol 3,350 and 0.2 M ammonium acetate (pH 7.1). The crystal appeared after about 2–3 days at 293 K and continued to grow up to a maximum size within 2 weeks.

### Data Collection and Structure Determination

Data for the caspase-8 DEDs were collected by the Pohang Light Source (PLS), Republic of Korea. Before data collection, the crystal was transferred to a cryoprotectant solution containing precipitant solution with 20%glycerol. After a brief soaking in the cryoprotectant solution, the crystal was flash-cooled to 100 K under liquid nitrogen stream. The diffraction data were processed using the *HKL*-2000 package [19]. The tertiary structure of the caspase-8 DEDs was determined by the molecular replacement method using the program *AMoRe* [20]. The model was improved by iterative model building using the program O [21]. The search was carried out using data obtained between 50.0 and 1.7 Å resolution (Table 1).

## Results and Discussion

**Fig. 1:**
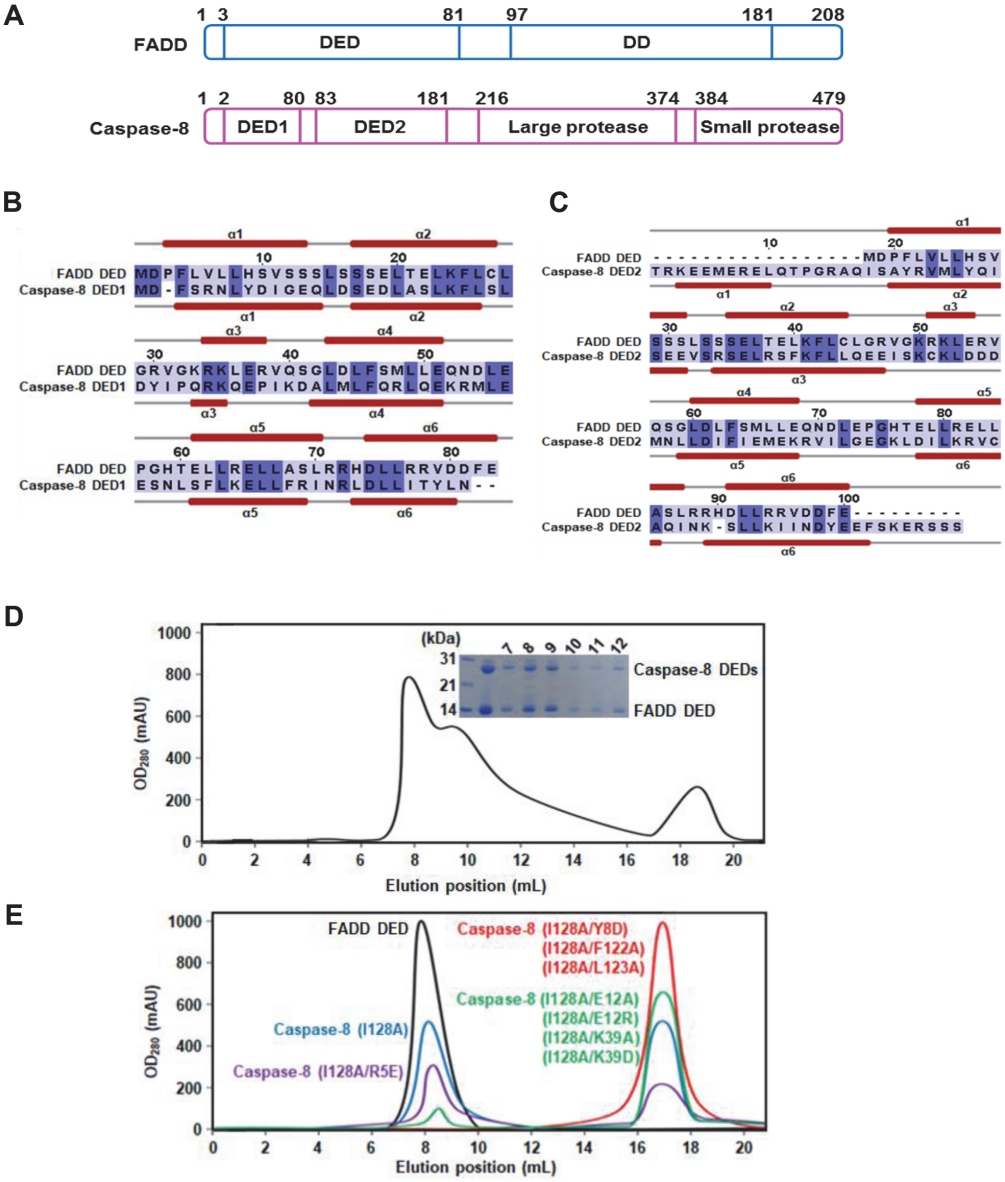
Domains and secondary structures of the FADD and Caspase-8. (**A**) Schematic representation of the fulllength FADD and Caspase-8. (B-C) Sequence alignments and secondary structures of FADD DED and Caspase-8 DEDs (DED1 and DED2) are shown. (**D**) Co-expression of FADD DED and Caspase-8 DEDs is shown by SDS-PAGE. Elution profile of the oligomeric DED complex is shown. (**E**) Gel-filtrations of FADD DED and Caspase-8 DEDs mutants are shown.

The domain structures of FADD and caspase-8 are shown in Fig. 1A. Human FADD is known as a mediator of receptor-induced toxicity 1 (MORT1). The full-length FADD protein contains the N-terminal DED (residues 3–81) and C-terminal DD (residues 97–181). The primary mediator of apoptosis, human caspase-8, exists in the cytoplasm as an inactive form composed of two structural homology domains (DED1, residues 2–80; DED2, residues 83–181), a large protease subunit (p18, residues 216–374), and a small protease subunit (p10, residues 384–479). It is converted to the active form through cleavage sites (residues Asp216, 374 and 384) (22). The amino acids sequence and secondary structure of the FADD DED (residues 1–83) were aligned with caspase-8 DED1 (residues 1–80) and DED2 (residues 81–188) and they contain only α-helices and loop regions (Figs. 1B and 1C).

To obtain a soluble FADD DED and caspase-8 DED complex, recombinant FADD DED and caspase-8 DEDs were isolated by co-expression. The soluble FADD and caspase-8 proteins were purified to homogeneity and their binding was detected using SDS-PAGE. We found a co-expression complex peak by FPLC, which indicated a molecular weight of over 200 kDa. (Fig. 1D). The FADD DED and caspase-8 DED complex band from the gel-filtration showed the formation of a high-order oligomeric DED complex.

To elucidate the key determinants in DED/DED interaction, point mutations of caspase-8 DED fusion protein were performed. The SWISS-MODEL was used to predict a homology model by structure databases and bridging the gap between sequences. The interaction between FADD DED and caspase-8 DEDs was modeled using our caspase-8 DED structure and the known structure of human FADD DED (PDB ID: 2GF5). The self-oligomerization or complex formation of FADD DED and caspase-8 DEDs (I128A and R5E/I128A) in vitro was shown using gel-filtration experiment. Meanwhile, the mutants of caspase-8 DEDs (Y8D/I128A, E12A/I128A, E12R/I128A, K39A/I128A, K39D/I128A, F122A/I28A, and L123A/I128A) did not exhibit self-oligomer formation (Fig. 1E).

**Fig. 2:**
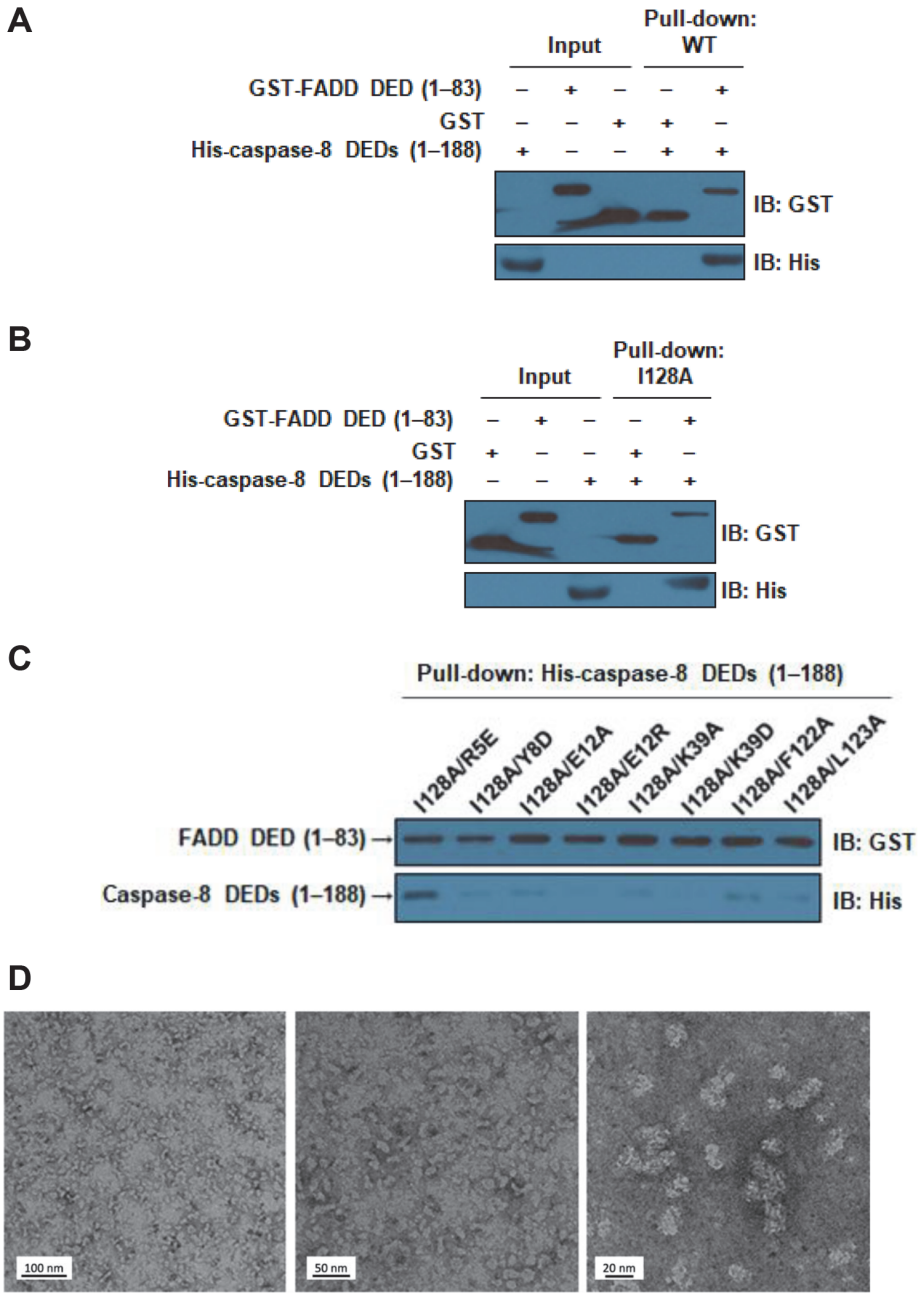
Interaction analysis of FADD DED and Caspase-8 DEDs using GST pull-down assay. (**A**) Interaction analysis of wild-type FADD DED and Caspase-8 DEDs using GST pull-down. (B and C) Interaction analysis of FADD DED and Caspase-8 DEDs (I128A) and Caspase-8 DEDs mutants using the GST pull-down. GST-tagged FADD was bound to glutathione-sepharose 4B beads. Seven mutants (I128A/Y8D, I128A/E12A, I128A/E12R, I128A/K39A, I128A/K39D, I128A/ F122A, and I128A/L123A) of Caspase-8 DEDs showed defective interaction. (**D**) Observation of FADD DED-Caspase-8 DEDs co-expression complex by EM is shown. Middle panel of Figure 2D was recorded at magnification of a high-resolution image (84,000X). The relative magnitudes of the electron magnification were shown.

The binding between FADD and caspase-8 was also identified using a GST pull-down assay (Figs. 2A-2C). The GST pull-down assay was performed using His-caspase-8 DEDs (1–188) with GST-FADD DED fusion protein. These results revealed that wild-type FADD DED interacts with caspase-8 DEDs in vitro. In addition, the four binding sites (R5, Y8, E12, and K39) are predicted in the modeled FADD DED-caspase-8 DED complex structures and three known mutation sites (F122, L123 and I128) are used. Wild-type FADD DED still interacts with mutant caspase-8 DEDs (I128A and R5E/I128A), respectively. However, the DED/DED interactions of seven mutations of caspase-8 DED (Y8D/I128A, E12A/I128A, E12R/I128A, K39A/I128A, K39D/I128A, F122A/I128A, and L123A/I128A) were disrupted by the GST pull-down assay. Especially, the mutants E12R and K39D showed low protein expression and no complex formation (Fig. 2C).

To define the molecular basis of DED interactions in the complex, we performed electron microscopy (EM) on the wild-type FADD DED and caspase-8 DED co-expression complex and its morphology is shown (Fig. 2D). A microscopic image of negatively stained hetero-oligomeric protein complex was recorded, and protein oligomer was shown to be homogeneous in overall shape.

**Fig. 3:**
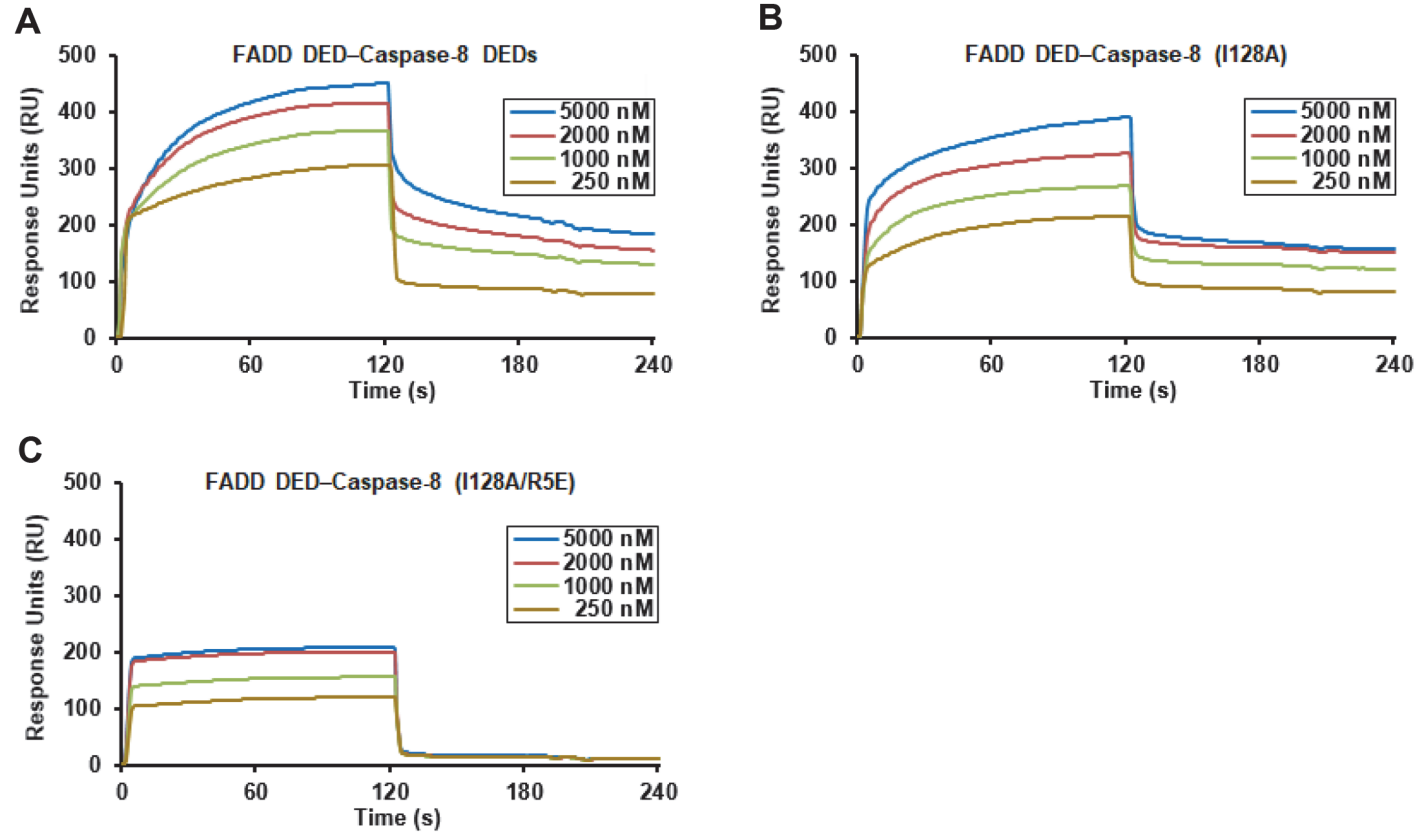
BIAcore biosensor analysis of interaction between FADD DED and Caspase-8 DEDs. (**A**) The binding of wild-type FADD DED and Caspase-8 DEDs is shown using BIAcore biosensor analyte. (**B-C**) Interaction analyses of FADD DED and Caspase-8 DEDs (I128A) and Caspase-8 DEDs (R5E/I128A) are shown using BIAcore biosensor at 25°C. The FADD and Caspase-8 sensorgrams for 250, 1,000, 2,000, and 5,000 nM are shown.

The binding affinity of caspase-8 DEDs for FADD DED was estimated using surface plasmon resonance (SPR) spectroscopy (Fig. 3). We found that wild-type caspase-8 DEDs physically bind strongly to wild-type FADD DED with an apparent *K*_D_ of 38 nM. The interaction is stronger than those between FADD DED and mutated caspase-8 DEDs (I128A or R5E/I128A) (*K*_D_ of 61 or 236 nM). Seven mutations including Y8, E12, K39, F122, and L123 residues disrupted FADD DED homotypic interaction with large *K*_D_ and negative RU values.

**Fig. 4:**
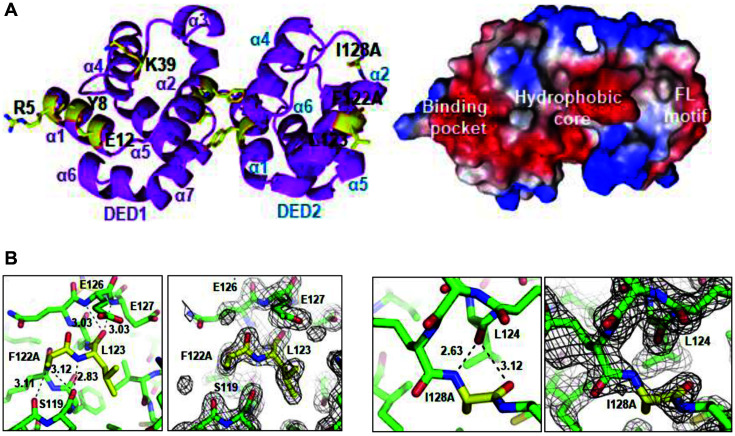
Crystal structure and electron density map of the Caspase-8 DEDs (F122A/I128A). (**A**) Ribbon diagram of the Caspase-8 DEDs (F122A/I128A) is shown with magenta color (left). The molecule of the Caspase-8 DEDs (F122A/ I128A) is shown as surface representation (right). Blue and red represent positive and negative electrostatic potentials, respectively. (**B**) Electron density map of the Caspase-8 DEDs (F122A/I128A) is shown. Red and blue represent oxygen and nitrogen atoms, respectively. Dotted line indicated the intermolecular hydrogen bonds. The mutated residue of Caspase-8 DEDs (F122A) and L123 of FL motif are shown (left panel). The mutated residue of Caspase-8 DEDs (I128A) is shown (right panel).

We attempted to make a crystal of the FADD DED and caspase-8 DED complex but were not successful. However, the crystal of mutant caspase-8 DEDs (1–188, F122A/I128A) was obtained using the hanging-drop vapor diffusion method. It was grown using a reservoir of 20% w/v polyethylene glycol 3,350 and ammonium acetate (pH 7.1) and belongs to the C2 space group. The diffraction data were collected at a high resolution of 1.7 Å. The statistics for the X-ray diffraction data collection are summarized (Table 1). The monomeric His-tagged caspase-8 DEDs (F122A/I128A) consist of only α-helices and loop regions and they have a dumbbell-shaped structure with rigidly associated DED1 and DED2 through hydrophobic interaction (Fig. 4). The interface amino acids (R5, Y8, E12, and K39) between FADD DED and caspase-8 DEDs are located on the binding pocket of the caspase-8 DEDs. The side chains for R5, Y8, E12, and K39 are exposed to DED1 of the caspase-8 surface. Other DED2 binding sites (F122A, L123 and I128A) of caspase-8 DEDs are located on the FL motif.

To investigate whether FADD interacts with caspase-8, truncated FADD DED and caspase-8 DEDs were characterized and purified as fusion *E. coli*. In the present study, co-expression, SEC, and Biacore biosensor analysis of the interaction between FADD DED and caspase-8 DEDs were performed. They support direct homotypic interactions between their DEDs. Moreover, these results indicate that several residues on the interface play a pivotal role in the assembly and regulation of homo- and hetero-complex formation of the death complex, which is crucial for understanding programmed apoptotic cell death mediated by FADD and caspase-8.

This study showed that mutations of some key binding residues (E12 and K39) in caspase-8 DED1 disrupted the interaction with FADD DED (Figs. 2 and 3). The interaction sites (Y8, E12, K39, F122, and L123) in caspase-8 are required for formation of DED homotypic interactions. Alteration of some DED residues in FADD, caspase-8, and Fas blocks receptor-mediated apoptotic cell death through several mechanisms [23]. Many extrinsic signaling pathways stimulate programmed cell death in tumor cells. These events are independent of tumor-suppressor protein p53 [24]. Thus, FADD and caspase-8 might be useful targets in cancer research studies. The present study provides crucial data on the structures of FADD and caspase-8 as well as the binding affinity of caspase-8 toward FADD, and finally, the homotypic interactions of DEDs. Our findings provide additional insight into the mechanisms underlying the interactions between DED superfamily proteins.

## Figures and Tables

**Table 1:** Crystallographic statistics.

Data collection statistics	
Crystal	Caspase-8 DEDs (F122A /I128A)
Space group	*C*2
Unit cell dimensions (Å)	a = 100.5, b = 51.2, c = 52.1 α = 90.0°, β = 116.3°, γ = 90.0°
Resolution (Å)	50.0-1.7 (1.73-1.70)
Completeness (%)	99.7 (100)
Observed reflections	477,168
Unique reflections	26,340
*I/σ* (*I*)	32.5 (2.6)
*R*_merge_ (%)^a^	5.2 (28.7)
Redundancy	3.8
Refinement statistics	
Resolution range (Å)	50-1.7
*R*_cryst_/*R*_free_ (%)^b^	22.7/25.4
Proteins/Water	183/143
Rmsd bond length (Å)/angles (°)	0.005/1.004
Average B-factor (Å^2^) Ramachandran plot (%)	47.9
Most favored region	93.1
Additional allowed region	6.9
Generously allowed region	0.0
Disallowed region	0.0

Values in parentheses are for the highest resolution shell. ^a^*R*_merge_ = Σ|*I*_i_ *I*_m_|/Σ*I*_i_, where Ii is the intensity of the measured reflection and *I*_m_ is the mean value of all symmetry-related reflections. ^b^*R*_cryst_ = S||*F*_obs_| - |*F*_calc_||/S|*F*_obs_|, where *F*_obs_ and *F*_calc_ denotes the observed and calculated structure factor amplitude. *R*_free_ = Σ*_T_*||*F*_obs_| - |*F*_calc_||/S*_T_*|*F*_obs_|, where T is a test data set of about 5% of the total reflections randomly chosen and set aside prior to refinement.
